# Comparison of the Filtration Culture and Multiple Real-Time PCR Examination for *Campylobacter* spp. From Stool Specimens in Diarrheal Patients

**DOI:** 10.3389/fmicb.2018.02995

**Published:** 2018-12-05

**Authors:** Hao Liang, Ziyu Wen, Ying Li, Yongxiang Duan, Yixin Gu, Maojun Zhang

**Affiliations:** ^1^State Key Laboratory for Infectious Disease Prevention and Control, National Institute for Communicable Disease Control and Prevention, Chinese Center for Disease Control and Prevention, Beijing, China; ^2^Nanshan Center for Disease Control and Prevention, Shenzhen, China; ^3^Shunyi District Center for Disease Control and Prevention, Beijing, China

**Keywords:** *Campylobacter*, culture, quadruple real-time PCR, diarrheal patients, comparison

## Abstract

*Campylobacter* is one of the most common pathogens leading to the bacterial diarrheal illness. In order to set up one effective culture independent assay for the screen of the *Campylobacter* infection in the diarrheal patients, the quadruple real-time PCR method comparing to the culture based on the enriched filtration method which was recognized as the most effective isolation method was assessed for 190 stool samples from the diarrheal patients collected during the Foodborne Diseases Active Surveillance Network in Beijing. This multiple real-time PCR was designed to identify the *Campylobacter* genus, *C. jejuni, C. coli*, and *C. lari* simultaneously. With the enrichment culture method, 23 (12.1%, 23/190) *Campylobacter* isolates were obtained (20 *C. jejuni* and 3 *C. coli*), however, 31 samples (16.3%, 31/190) were detected positively with the real-time PCR (21 *C. jejuni*, 8 *C. coli*, and 2 *Campylobacter* genus only). With the comparison, the real-time-PCR method is more sensitive than the enrichment filtration method (16.3 vs. 12.1%, *p* = 0.021). Among the culture-positive samples, 95.7% (22/23) were detected positively by PCR which indicate the specificity of this method was higher. These two methods were consistent well (Kappa = 0.785, *p* < 0.05). Comparing to the culture methods, the result of the multiple real-time PCR method is sensitive, reliable and rapid. The present study indicated this multiple real-time PCR can be used both for the surveillance network and the preceding screen for bacteria isolation. This is first comparative study between the culture and multiple real-time PCR method for *Campylobacter* identification in stool specimens from the diarrheal patients.

## Introduction

Foodborne *Campylobacter* is recognized as the leading causes of the bacterial diarrheal illness in both developing and developed countries (Coker et al., 2002; EFSA, 2015; Marder et al., 2017; Li et al., 2018; Natsos et al., 2018). One recent report showed that the isolation ratio of *Campylobacter* ranked the third among the major bacterial pathogen in the diarrheal cases in Beijing (Li et al., 2018).

Bacteria culture has been recognized as the “Gold standard” for the diagnosis of the *Campylobacter* infection (de Boer et al., 2015). However, the isolation result could be affected with many factors including the culture method and the medium. Culture-independent diagnostic tests (CIDTs) are increasingly used to diagnose *Campylobacter* infection in the Foodborne Diseases Active Surveillance Network (FoodNet) in United States and other countries (Bessède et al., 2011). The incidence of the culture confirmed and CIDT positive infections was highest for *Campylobacter* among bacterial and parasitic infections (confirmed = 11.79; CIDT positive–only = 17.43) per 100,000 persons in United States (Marder et al., 2017).

In order to explore the effective methods used for *Campylobacter* surveillance in China, in this study, we compared the quadruple real-time PCR method which could simultaneously identify the *Campylobacter* genus and three major foodborne *Campylobacter* spp., *C. jejuni, C. coli*, and *C. lari* to the culturing method from the stool specimens collected from the diarrheal patients in the Foodborne Diseases Active Surveillance Network (FoodNet) in Shunyi District, Beijing.

## Materials and Methods

### Sample Collection and Patients

A total of 190 stool samples from diarrhea patients were collected in Shunyi and Konggang Hospitals in Shunyi district, Beijing from July 2017 to February 2018. 190 specimens were collected from 190 cases. Among the 190 cases, 121 cases were male and 69 cases were female. The age of the patients ranged from 15 to 85 years old. The clinical character of the patients were summarized in Table 1. Five mg fresh stool samples were collected from each patient and transported in Cary-Blair medium at 4°C to the laboratory within 24 h.

**Table 1:** Clinical characteristics of the entire tested cases and the characteristics of the culture-positive cases of each pathogen.

Clinical symptoms	*Campylobacter positive* (Pn/Tn)^a^	*Salmonella positive* (Pn/Tn)	DEC positive (Pn/Tn)	*Vibrio parahaemolyticus positive* (Pn/Tn)	Total (Pn/Tn)	*c*^2^	*p*-value
Abdominal pain	17/23	8/9	4/7	10/16	138/190	2.699	0.462
Nausea and vomiting	19/23	8/9	4/7	12/16	146/190	2.667	0.461
Dehydration	18/23	8/9	6/7	12/16	146/190	0.809	0.926
Feeling thirsty	12/23	3/9	5/7	12/16	88/190	4.81	0.174
Fever	9/23	5/9	1/7	2/16	51/190	6.416	0.087
Feeling weak	19/23	7/9	6/7	15/16	131/190	1.756	0.719

*^*a*^Pn, number of positive samples; Tn, number of total samples*.

### Bacteria Isolation and Identification

*Campylobacter* isolation was performed using the *Campylobacter* isolation kit (ZC-CAMPY-002, Qingdao Sinova Biotechnology Co., Ltd., Qingdao, China) according to the manufacturer’s instructions (Li et al., 2018) and the suspected isolates were picked on the Karmali agar in a microaerophilic atmosphere (5% O_2_,10% CO_2_, and 85% N_2_) at 42°C for 48 h. The species of *Campylobacter* were identified by multiple PCR (Wang et al., 2002). Meanwhile, the isolation for other four bacterial pathogens were also performed such as *Salmonella, Shigella*, Diarrheagenic *E. coli* [DEC, including *Escherichia coli* (EPEC), *enterotoxigenic E. coli* (ETEC), *enterohemorrhagic E. coli* (EHEC), *enteroinvasive E. coli* (EIEC), and *enteroaggregative E. coli* (EAEC)] and *V. parahaemolyticus* by traditional culture methods as described previously (Li et al., 2018).

### DNA Extraction and the Quadruple Real-Time PCR Analysis

The DNA of the stool samples were extracted using the QIAamp DNA Stool Mini Kit (Qiagen) according to the manufacturer’s protocol. All of the DNA samples were triplicate and stored at -80°C until use. The quadruple real-time PCR commercial kit (Qingdao Sinova Biotechnology Co., Ltd., Qingdao, China) was used for detecting *Campylobacter* genus, *C. jejuni, C. coli*, and *C. lari*. As the description of manufacturer’s instructions, the primers and fluorophore-linked probes for genus *Campylobacter* were targeted on the 16s rRNA gene, which can detect the major foodborne *Campylobacter spp.* including *C. jejuni, C. coli, C. lari, C. upsaliensis*, and *C.* *hyointestinalis.* Moreover, the target genes of *C. jejuni, C. coli*, and C. *lari* are *gyrA, ceuE*, and *glyA*, respectively. We used a real-time PCR commercial kit in this study so the exact primer and probe sequences are not available. The fluorescence labeled for the probes of the *Campylobacter* genus, *C. jejuni, C. coli*, and *C. lari* were FAM, HEX, Cy5, and Quasar 705 with the quencher dye BHQ1, BHQ1, BHQ3, and BHQ3, respectively.

The quadruple real-time PCR was carried out on a CFX96 (Bio-Rad, United States) thermal cycler, with the TransStartTM Probe real-time PCR SuperMix (TransGen Biotech, China). For this real-time PCR, a 20 μl reaction was performed contained 3 μl of template DNA, 10 μl TransStartTM Probe real-time PCR SuperMix, 0.3 μmol l^-1^ of each amplification primer and 0.15 μmol l^-1^ of each probe. The thermal cycle protocol consisted of initial denaturation at 94°C for 30 s, followed by 40 cycles of 94°C for 15 s and 60°C for 40 s. The result data of real-time PCR was analyzed with CFX manager software (version 2.0). Quantitative results of real-time PCR were based on log_10_ value transformed and threshold cycle (Ct). All the samples with Ct < 30 were considered positive. The Ct > 35 was considered as negative and 30 < Ct < 35 was defined as suspected with three times repeat.

The three species of *Campylobacter* strains stored in our laboratory were used as the positive reference strains. Several kinds of other genus bacteria stored in our laboratory was used as negative controls such as *Helicobacter pylori, E. coli, Streptococcus pneumoniae*, etc.

### Statistical Analysis

Significance of differences (*p* < 0.05) between culture and the real-time PCR was analyzed using chi-squared test and correlations between culture and detection was tested using Kappa test. The statistical analysis was performed using SPSS (version 22.0).

### Ethics Statement

There was no tissue or blood samples were involved in this study. All the bacteria were isolated from stool specimens from the patients. The purpose of the study was informed to the participants and written consent was given to all of them. This project was approved by the ethics committee of the China CDC and the academic committee in the National Institute for Communicable Disease Control and Prevention. All relevant documents were recorded in China CDC (ICDC-2014012).

## Results

### Bacterial Isolation

Among the 190 specimens, 54 samples were positive for the tested five major enteric pathogens and two of them were mixed infection. In one sample, *C. jejuni* and ETEC were isolated and in another one, *C. coli* and *Shigella* were isolated. *Campylobacter* (12.1%, 23/190) was the most common pathogen. 20 samples (10.5%, 20/190) were positive for *C. jejuni* and 3 (1.5%, 3/190) samples were positive for *C. coli*. The isolation of other pathogens was listed in Table 2. Nine samples were *Salmonella* positive, one *Shigella* isolate was obtained from 1 sample, 16 samples were *Vibrio parahaemolyticus* positive and 7 samples were DEC positive, respectively. The clinical symptoms of *Campylobacter* infection patients are not statistically different (see Table 1, *p* > 0.05) with other pathogens infection.

**Table 2:** Pathogen culture results of the 190 stool samples.

Pathogen	% (Pn/Tn)
*Salmonella*	4.7 (9/190)
*Shigella*	0.5 (1/190)
*Vibrio parahaemolyticus*	8.4 (16/190)
DEC	3.7 (7/190)
*Campylobacter*	12.1 (23/190)
Total	29.5 (56/190)

### Real-Time PCR Results and Comparison With Bacterial Culture

The entire 190 samples were performed by real-time PCR to detect the genus and identify the species of *Campylobacter* simultaneously. The results of the real-time PCR are shown in Table 3. Twenty-one *C. jejuni* (11%, 21/190) and eight *C. coli* (4.2%, 8/190) were detected in all samples. Among the 21 *C. jejuni* PCR positive samples, 18 (86%, 18/21) of them were culture positive but only 3 of the 8 *C. coli* PCR positive samples were culture positive (37.5%, 3/8). Only one culture-positive sample was not detected by this PCR method. There were two samples had positively result only on the genus *Campylobacter* but negative for the tested three species, which indicated the possibility of the infection with *C. upsaliensis* and *C.* *hyointestinalis* and need further confirmation.

**Table 3:** Comparison between culture and real-time PCR for *Campylobacter* detection.

*Campylobacter* species	Culture % (Pn/Tn)	Real-time PCR % (Pn/Tn)	*p*-value	Kappa
*Campylobacter* genus	12.1 (23/190)	16.3 (31/190)	0.021	0.785
*C. jejuni*	10.5 (20/190)	11.1 (21/190)	1	0.864
*C. coli*	1.6 (3/190)	4.2 (8/190)	0.063	0.535
*C. lari*	0	0	-	-
Total	12.1 (23/190)	16.3 (31/190)	0.021	0.785

**FIGURE 1:**
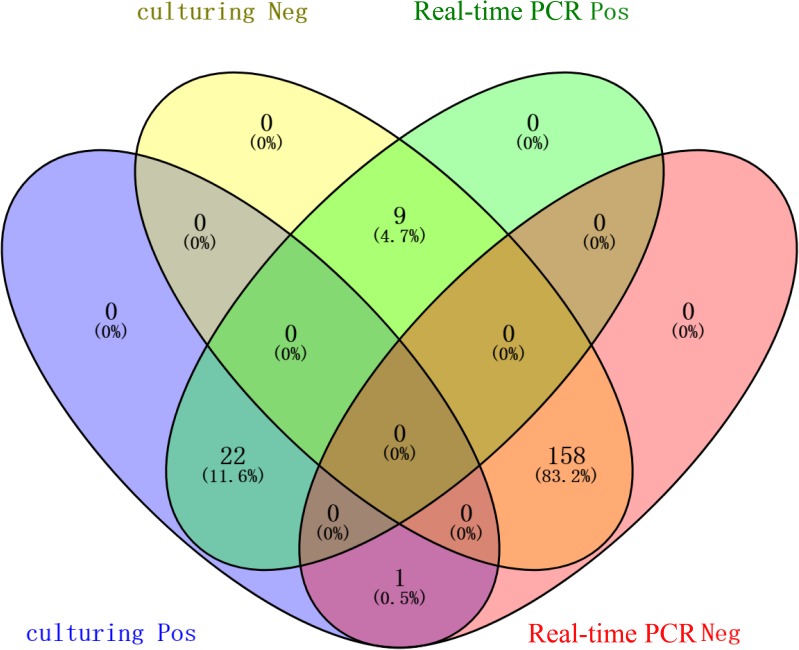
Venn-diagram summarizing the results of the culturing and the multiple real-time PCR detection method. The numbers in blue modules represent the number of culturing positive samples. The numbers in light yellow modules represent the number of culturing negative samples. The numbers in pink modules represent the number of real-time PCR negative samples. The numbers in green modules represent the number of real-time PCR positive samples.

The comparison of the PCR and the culturing results is shown in Figure 1. The coherence analysis of these two methods was Kappa = 0.785 (*p* < 0.05), which means the two methods are consistent well. According to the result of chi-squared test, the two methods in detecting *Campylobacter* are statistically different (*p* = 0.021) and the positive ratio of real-time PCR is higher than the culture (16.3 to 12.1%). For *C. jejun*i and *C. coli*, the difference of these two methods were not statistically significant (*p* = 1 and *p* = 0.063, respectively).

## Discussion

Bacterial diarrhea is a serious public health problem worldwide, especially *Campylobacter* spp. considered as a leading cause (Lynch et al., 2011; Wei et al., 2014; Nohra et al., 2016; Pavlova et al., 2016). In this study, the isolation of *Campylobacter* is also being the most common pathogen among the tested five enteric bacterial pathogens with the ratio of 12.1% (23/190). The *Campylobacter* isolation from the diarrheal patients using selective medium is variable. The considerable difference of the infection ratio in these reports was observed (Zhu et al., 2016; Chang et al., 2017; Zhang et al., 2017). Picking out the suspected *Campylobacter* colonies on the selective medium was laboratory experience depending and become more and more difficult with the increased drug-resistant microorganisms in the samples which reduced the selection capacity of the selective medium in these days in China. The enrichment with filtration method was recognized as the most effective isolation method for *Campylobacter* from the stool samples from the diarrheal patients (Li et al., 2018).

The use of CIDTs is increasing in the surveillance network in the developed counties (Cronquist et al., 2012; Babady et al., 2018). Stool antigen tests for *Campylobacter* from the stool samples was rapid but still with the problems of specificity and positive predictive value (Giltner et al., 2013). The method of multiple real-time PCR is widely used in detecting and identifying *Campylobacter* (Rudi et al., 2004; Ivanova et al., 2014). In this study, we used quadruple real-time PCR method which can detect the major foodborne *Campylobacter* spp. in one tube simultaneously. Comparing to the filtration method (culture with enrichment), this quadruple real-time PCR is more sensitive and reliable (16.3 to 12.1%, *p* = 0.021) and the whole PCR process was only take about 40 min to get the result. In this study, the *Campylobacter* was detected as the most common pathogen among the 190 diarrheal patients, which was consisting with the result of the FoodNet in United States (Marder et al., 2017).

Among the 31 PCR positive samples (16.3%, 31/190), the isolation rate is 71.0% (22/31). In addition to the sensitivity problem of the method (de Boer et al., 2015), there were other reasons may cause the result of 9 samples uniquely had PCR positive: the quality of the samples or the proceed of the sample transport caused the *Campylobacter* in a non-cultivable state. Certainly, the false positive results of the PCR might happened. PCR detection has the capacity to detect the DNA of dead bacteria or non-cultivable bacteria which could also indicated the recent infection. Only one sample was culture positive with *C. jejuni* but the CT value of the genus was more than 35 with three times repeats. The reason might be the amount of the pathogen is not enough or there was lost during the DNA extraction.

There are numbers of previous study indicated that the sensitivity of the real-time PCR was less than the culture method (Lund et al., 2004; Hong et al., 2007; Mily et al., 2011). Our study indicated the two methods for each of *C. coli* and *C. jejuni* is agreed with almost perfect agreement (Kappa = 0.864) and moderate agreement (Kappa = 0.535), respectively, and the difference was not statistically significant. This is first comparative study between the culture and multiple real-time PCR method for *Campylobacter* identification in stool specimens from the diarrheal patients.

To date, the surveillance for *Campylobacter* infection in the diarrheal patients were only carried out in parts of China. According to some previous publication (Zhu et al., 2016; Chang et al., 2017; Zhang et al., 2017), the campylobacteriosis in China was low and it might because of the detection method was inefficient. From this study, the results using the multiple- real time PCR indicated the *Campylobacter* infection accounts for a high proportion in diarrheal patients in Beijing. With the effective screening method, we could get the reliable data more quickly and it will specifically benefit the food safety assessment plan in China.

In conclusion, with the comparison of the real-time PCR method to the bacteria culture with enrichment in the *Campylobacter* detection from the stool sample, this quadruple real-time PCR method is a reliable and sensitive. This method is suitable to be used as a rapid screening method. Besides, the result can be used as guide for the pathogen culture which can get the isolates in the surveillance work in the future.

## Author Contributions

HL, YD, ZW, and YG performed the molecular test and data analysis. YL was involved in the collection of samples and DNA extraction. HL and MZ drafted and revised this manuscript.

## Conflict of Interest Statement

The authors declare that the research was conducted in the absence of any commercial or financial relationships that could be construed as a potential conflict of interest.
